# Microbes and pain

**DOI:** 10.1371/journal.ppat.1009398

**Published:** 2021-04-01

**Authors:** Liwen Deng, Isaac M. Chiu

**Affiliations:** Harvard Medical School, Blavatnik Institute, Department of Immunology, Boston, Massachusetts, United States of America; Duke University School of Medicine, UNITED STATES

## Nociceptors, microbes, and pain

Microbial infections are often painful. Pain is a defense mechanism that alerts organisms to threats and is mediated by nociceptors, first described by Charles Sherrington in 1906 as the sensory afferent neurons that induce withdrawal reflexes in response to noxious stimuli [1]. Nociceptors densely innervate barrier tissues that interface with the environment, including the skin, lungs, urinary tract, and gut, and deeper tissues such as the joints, bones, and meninges. Nociceptor neurons express receptors such as transient receptor potential (TRP) channels that are gated to detect noxious/harmful stimuli including heat, cold, and reactive chemicals [2]. Upon sensing a damaging stimulus, action potentials are transmitted to nociceptor cell bodies in the dorsal root ganglia (DRG), which receive input from peripheral tissues such as the skin. Related sensory neurons in the vagal ganglia receive input from internal organs such as the heart, gut, and lungs. DRG signals are sent to the spinal cord to be perceived as pain, while vagal signals are sent to the brain stem and mediate nausea, cough, and other protective reflexes.

Nociceptors, like immune cells, play an active role in host–pathogen defense. Nociceptors express receptors including Toll-like receptors (TLRs) and formyl peptide receptors (FPRs) that can directly sense microbes. Furthermore, nociceptors release neuropeptides that potently signal to immune cells. We review recent studies that highlight how microbes interact with nociceptors during infection to modulate pain and cough.

## Bacterial pathogens, pain, and cough

**Fig 1:**
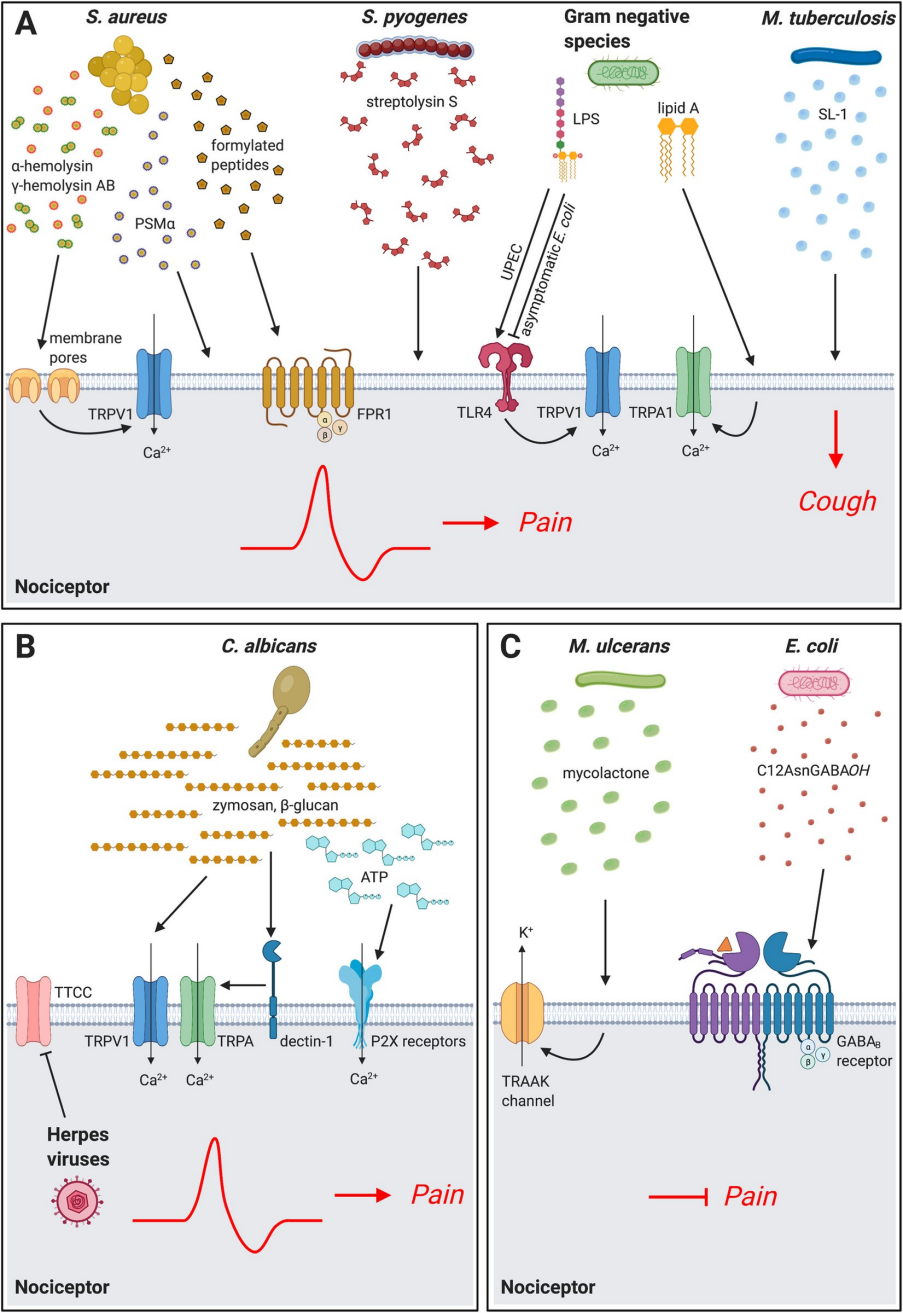
Microbial mechanisms that act on nociceptor neurons to mediate pain and cough. **(A)** Bacterial pathogens directly activate nociceptors to cause pain and cough during infection. *S*. *aureus* produces the pore-forming toxins α-hemolysin, bicomponent leukocidin HlgAB, and PSMα, which act on sensory neurons to induce cation influx, action potential generation, and pain production. *S*. *aureus* formylated peptides bind to FPR1 receptors on nociceptors to induce mechanical pain sensitivity. *S*. *pyogenes* produces the cytolytic toxin SLS to activate nociceptors and produce pain during infection. LPS from gram-negative bacteria including UPEC can act through TLR4 expressed on nociceptors to sensitize the TRPV1 ion channel. LPS from several gram-negative bacterial species also directly activates the nociceptive ion channel TRPA1, an activity that depends on the lipid A moiety. By contrast, LPS from asymptomatic *E*. *coli* can block pain. *M*. *tuberculosis* produces SL-1, which induces calcium influx in lung-innervating nociceptors and mediates cough during infection. **(B)** Fungal and viral mechanisms of pain. *C*. *albicans* cell wall components zymosan and β-glucan can activate neurons to produce pain. β-glucan can directly activate neurons via the dectin-1 receptor expressed by nociceptors. β-glucan also stimulates keratinocytes to release ATP, which subsequently activates P2X receptors on neurons to produce pain. Herpesviruses infect nociceptor neurons, reducing the expression of TTCC, which alters electrical excitability of neurons. **(C)** Microbial products that silence neurons and pain. *M*. *ulcerans* alters nociceptor signaling through a mycolactone that induces potassium efflux via TRAAK family potassium channels. *E*. *coli* Nissle 1917 secretes the C12AsnGABA*OH* lipopeptide which inhibits nociceptor activation and pain via the GABA_B_ receptor. Created with BioRender.com. FPR, formyl peptide receptor; LPS, lipopolysaccharide; PSMα, phenol soluble modulin α3; SL-1, sulfolipid-1; SLS, streptolysin S; TLR, Toll-like receptor; TRP, transient receptor potential; TTCC, T-type calcium channels; UPEC, uropathogenic *Escherichia coli*.

A number of bacteria can activate nociceptors to cause pain and cough during infection (Fig 1A). *Staphylococcus aureus*, an important human pathogen, possesses a variety of virulence factors that facilitate host invasion. Of these virulence factors, *S*. *aureus* pore-forming toxins (PFTs), which bind to membrane receptors on host cells and oligomerize to produce transmembrane pores, have been demonstrated to directly activate nociceptors. The PFTs α-hemolysin, phenol soluble modulin α3, and bicomponent leukocidin HlgAB are sufficient to induce firing by DRG neurons and pain in mice [3,4]. In a mouse model of *S*. *aureus* infection, α-hemolysin was necessary for both acute pain and hyperalgesia during infection [3,4]. *S*. *aureus* also secretes N-formyl peptides, which mediate mechanical pain during infection through activation of FPR1 expressed by nociceptors [3]. *S*. *aureus* α-hemolysin activates a broad group of nociceptor neurons which express the Nav1.8 sodium channel and the heat-sensitive ion channel TRPV1. N-formyl peptides act on a smaller subset of nociceptors which respond to both capsaicin, a TRPV1 ligand, and allyl isothiocyanate, a TRPA1 ligand. While α-hemolysin contributes to both mechanical and thermal pain sensitivity, N-formylated peptides only contribute to mechanical sensitivity [3].

*Streptococcus pyogenes* is a causative agent of infections characterized by intense pain such as pharyngitis (strep throat), cellulitis, and necrotizing fasciitis. While necrotizing fasciitis can be challenging to diagnose clinically, “pain out of proportion to physical exam” is considered a classic feature of the disease and has a strong predictive value [5]. *S*. *pyogenes* is its leading cause, and therefore, the molecular mechanisms of pain during infection are potentially clinically relevant. *S*. *pyogenes* M1T1 and M3 strains were found to directly activate mouse nociceptors to produce pain through the toxin streptolysin S (SLS), and this pain occurred independent of neutrophils, T and B cells, and other immune signaling molecules including MyD88 [6]. *S*. *pyogenes* directly induced calcium influx in TRPV1+ nociceptor neurons in an SLS-dependent manner. In mice, subcutaneous infection with *S*. *pyogenes* strains caused spontaneous pain and mechanical and thermal hyperalgesia, while mice infected with an SLS-deficient strain exhibit reductions in all 3 pain parameters; a neutralizing antibody against SLS also blocked spontaneous pain during infection [6]. TRPV1+ nociceptors release the neuropeptide calcitonin gene-related peptide (CGRP) from their peripheral terminals, which directly inhibited bactericidal killing of *S*. *pyogenes* and decreased myeloperoxidase activity of neutrophils. Local injection of botulinum neurotoxin a (BoNT/A), which blocks neurotransmission, or systemic treatment of mice with BIBN4096, a CGRP receptor antagonist, enhanced host defenses and *S*. *pyogenes* bacterial clearance [6]. Therefore, *S*. *pyogenes* may hijack a neuro-immune suppression mechanism to facilitate their survival.

Nociceptor modulation of immune cells through CGRP also occurs for *S*. *aureus* and other pathogenic infections. Neutrophils, macrophages, and dendritic cells express RAMP1 and CALCRL, which form the receptor for CGRP. CGRP signaling through RAMP1/CALCRL induces activation of ICER, which shuts down NF-κB signaling in macrophages and dendritic cells to modulate cytokine production [7,8]. In a mouse model of *S*. *aureus* pneumonia, TRPV1+ nociceptors inhibited both TNF-α and CXCL1 production in the lung through CGRP [9]. CGRP inhibits macrophage production of TNF-α in response to lipoteichoic acid or heat-killed *S*. *aureus* [3]. By contrast, activation of TRPV1+ nociceptors and release of CGRP have been shown to enhance epicutaneous dendritic and Type 17 immune cell responses against *Candida albicans* and *S*. *aureus* [10,11]. It is possible that the anatomical site of action (e.g., skin, gut, or lungs), route of infection (e.g., epicutaneous or subcutaneous), types of neurons involved (e.g., peptidergic or non-peptidergic), and target immune cell type (neutrophil or dendritic cell) could impact whether nociceptors lead to activation or suppression of immunity.

Nociceptors have also been shown to express TLR4 [12], the receptor for the gram-negative bacterial cell wall component lipopolysaccharide (LPS). Recently, it has been reported that LPS can directly activate nociceptors through TLR4. LPS treatment induces CGRP release from vagal ganglia, but ganglia from mice that lack TLR4 specifically in Nav1.8 nociceptors do not produce CGRP after LPS application [13]. TLR4 is necessary for pain induced by uropathogenic *Escherichia coli* (UPEC). Instilling LPS purified from the UPEC strain NU14 into the bladders of mice was sufficient to induce pelvic pain, which was significantly reduced in TLR4-deficient animals. By contrast, purified LPS from the asymptomatic bacteriuria *E*. *coli* strain 83972 did not cause pain in mice, suggesting distinctions between types of LPS in pain production [14].

LPS can also stimulate nociceptors in a manner dependent on the large-pore cation channel TRPA1. LPS induced calcium influx and membrane depolarization of DRG neurons, which was abolished in TRPA1 deficient cells, but independent of TLR4. Purified *E*. *coli* LPS induced acute pain responses and mechanical hyperalgesia in wild-type and *Tlr4*−/− mice, while these responses were reduced in *Trpa1*−*/*− mice. The effect of LPS on TRPA1 is dependent on the lipid A moiety, which produces mechanical alterations to the plasma membrane. How membrane perturbations can lead to TRPA1 activation is still unclear; however, the activity of lipid A is correlated with its shape. LPSs from *E*. *coli*, *Salmonella typhimurium*, and *Klebsiella pneumoniae* induced the strongest TRPA1 responses, whereas LPSs from *Serratia marascens*, *Pseudomonas aeruginosa*, *Neisseria meningitidis*, and *Salmonella minnesota* induced smaller or no TRPA1 responses [15].

Cough, a protective reflex in the respiratory tract that is analogous to pain, is mediated by nociceptor neurons residing in the vagal sensory ganglia that innervate the lungs. Pulmonary *Mycobacterium tuberculosis* (Mtb) infection often manifests with a persistent cough, which could facilitate its transmission. It was recently found that a key mediator of Mtb-induced cough is the Mtb cell wall glycolipid sulfolipid-1 (SL-1) [16]. In a guinea pig model of pulmonary Mtb infection, wild-type Mtb induced chronic cough, while SL-1–deficient Mtb did not. Mtb extracts and purified SL-1 were also found to directly activate mouse and human DRG and vagal nociceptor neurons. Treating healthy guinea pigs with nebulized SL-1 was sufficient to stimulate coughing. Because SL-1 is not a virulence determinant for Mtb (SL-1–deficient Mtb strains are equally as pathogenic as SL-1–producing strains), this work suggests that the bacterium may modulate nociceptors to produce a host behavior which could aid in its transmission to other hosts [16].

## Fungi and viruses that cause pain

In addition to bacteria, other microbes such as fungi and viruses can produce pain during infection (Fig 1B). The opportunistic fungal pathogen *C*. *albicans* can cause painful infections of several tissue sites including the skin. Calcium imaging experiments showed that heat-killed *C*. *albicans* and zymosan, a mixture of glucans present in fungal cell walls, can activate TRPV1-positive nociceptor neurons. DRG neurons also released CGRP in response to stimulation with *C*. *albicans*, which then signaled to dendritic cells to produce the cytokine interleukin (IL)-23 to stimulate IL-17 production from γδ T cells and activate an effective immune response to the fungus [10].

β-glucan, a component of the *C*. *albicans* cell wall, mediates fungal pain through activation of dectin-1, the receptor for β-glucan. Injecting *C*. *albicans* into the hind paws of mice induced spontaneous pain behaviors (licking of the paw) and mechanical hypersensitivity. Nociceptors express dectin-1 and can directly detect β-glucan through a downstream TRP channel–dependent mechanism; both dectin-1–deficient and TRPV1/TRPA1 double-deficient mice fail to develop mechanical allodynia after β-glucan treatment [17]. Keratinocytes also express dectin-1 and contribute to pain by releasing ATP that activates P2X receptors on nociceptor neurons [17].

The 3 major herpesviruses in humans, varicella zoster virus (VZV), herpes simplex virus 1 (HSV1), and HSV2, are well-known causes of neuropathic pain. These neurotropic viruses enter the host by infecting mucocutaneous surfaces where they access sensory nerves from trigeminal ganglia and DRG. Herpesviruses establish latent infection in nociceptors and, upon their reactivation, the virus can cause significant pain such as in postherpetic neuralgia [18]. HSV-1 may affect pain sensory transmission by reducing expression of T-type calcium channels and therefore altering electrical excitability in a sensory neuron–like cell line [19].

## Microbes that silence pain

While pain is typically considered a hallmark of infection, some pathogens can block pain during their disease cycle (Fig 1C). The bacterial pathogen *Mycobacterium ulcerans* causes persistent and painless skin lesions, and painlessness often occurs at early phases of skin infection, prior to the development of nerve damage. The *M*. *ulcerans* polyketide mycolactone has been shown to play a role in silencing pain. Mice infected with *M*. *ulcerans* exhibit hypoesthesia, and mycolactone injections were sufficient to induce analgesia. Application of mycolactone to neuronal cell cultures induced hyperpolarization that is mediated by potassium channels. Mycolactone-induced hyperpolarization could be inhibited by silencing phospholipase A2, which mediates synthesis of arachidonic acid, and cyclooxygenase-1, which metabolizes arachidonic acid into prostaglandin E2 (PGE_2_). Inside-out patch clamp experiments demonstrated that PGE_2_ mediates the release of potassium through the TRAAK subfamily of potassium channels [20]. An siRNA library and mouse knockout studies led to the conclusion that type 2 angiotensin II receptor (AT_2_R) was necessary for mycolactone-induced hyperpolarization. It is important to note that the effect of mycolactone signaling through neuronal AT_2_R on pain is controversial. Some studies describe antinociceptive effects of both AT_2_R agonists and antagonists [21]. Furthermore, recent single-cell transcriptomic studies reveal low or no expression of AT_2_R by nociceptors [22]. Therefore, future studies are needed to clarify the mechanisms by which *M*. *ulcerans* and mycolactone block pain.

Some strains of *E*. *coli* also have analgesic effects. The probiotic *E*. *coli* strain Nissle 1917 secretes a lipopeptide, C12AsnGABA*OH*, which can reduce visceral pain associated with irritable bowel syndrome through GABA_B_ receptor. C12AsnGABA*OH* prevented nociceptor activation by both the TRPV1 agonist capsaicin and a mixture of G protein–coupled receptor agonists (histamine, serotonin, and bradykinin). The inhibitory effect of C12AsnGABA*OH* was abolished by treatment with an antagonist of the GABA_B_ receptor. Intracolonic injection of C12AsnGABA*OH* prevented capsaicin-induced hypersensitivity in mice [23].

## Concluding remarks and future directions

It is now clear that pathogens can directly activate nociceptors to alter pain signaling and that pain participates in host defense. Defining the contributions of immune and pathogen activation of neurons is important for a more complete understanding of the causes of pain during infection. Cytokines, including interferons, IL-1, and TNF, can sensitize neurons and cause pain [24,25]. Recent transcriptome data show that nociceptors express other receptors for microbial products such as retinoic acid-inducible gene-I-like (RIG-I-like) receptors (RLRs), nucleotide-binding oligomerization domain-like (NOD-like) receptors (NLRs), and other TLRs, but their role during infection is not yet known. One interesting study showed that TLR5 is expressed by A-fiber nociceptors, which, upon activation with flagellin in combination with the charged analgesic QX-314, can block neuropathic pain [26]. However, the endogenous role of TLR5 in bacteria-induced pain has not been studied. Nociceptors also actively regulate immunity in both skin and mucosal tissues through neuronal mediators. For example, nociceptors suppressed immune responses during subcutaneous infection by *S*. *pyogenes* [6] and subcutaneous and lung infection by *S*. *aureus* [3,9], while inducing T cell immunity against *C*. *albicans* epicutaneous infection [10,11]. It is possible that neurons elicit specific immune responses at different anatomical sites, and the outcome of nociceptor activation may depend on the route of infection. Defining how nociceptor neurons signal to immune cells is a key question in neuroimmunology that will require combinations of neurobiological, microbiological, and immunological investigations. Therapeutically, identifying molecular mechanisms by which neurons are modulated by microbes could lead to novel treatments of pain and infection.
